# Pituitary Apoplexy Following Gonadotropin‐Releasing Hormone Agonist Administration for Prostate Cancer

**DOI:** 10.1002/iju5.70159

**Published:** 2026-02-27

**Authors:** Masaaki Fujimura, Takahiro Shimizu, Shinichi Sakamoto, Hiroaki Sato, Hiroyoshi Suzuki, Kazuo Mikami

**Affiliations:** ^1^ Department of Urology Chibaken Saiseikai Narashino Hospital Chiba Japan; ^2^ Department of Urology Chiba University Chiba Japan; ^3^ Department of Urology Toho University Medical Center Sakura Hospital Chiba Japan

**Keywords:** gonadotropin‐releasing hormone agonist, pituitary apoplexy, prostate cancer

## Abstract

**Introduction:**

Pituitary apoplexy represents an uncommon endocrine emergency with potentially life‐threatening consequences. Gonadotropin‐releasing hormone agonist used for prostate cancer has the potential to induce pituitary apoplexy, particularly in the setting of a preexisting pituitary adenoma.

**Case Presentation:**

A 76‐year‐old male with prostate cancer initially chose active surveillance; however, prostate‐specific antigen (PSA) elevation required hormonal therapy 5 years later. A pancreatic islet tumor had been previously identified; however, its details were unavailable. He was presented to the emergency department 24 h after receiving the first gonadotropin‐releasing hormone agonist injection. He showed severe headache, general fatigue, diplopia, and ptosis of the right eye. Brain MRI revealed a right deviated suprasellar pituitary adenoma with hemorrhagic infarction. He was conservatively treated with high‐dose steroids; symptoms improved within 1 week.

**Conclusion:**

Clinicians should be aware of the association of pituitary apoplexy with the use of gonadotropin‐releasing hormone agonist and should ask about the patient's past history of multiple endocrine neoplasia (MEN).

## Key Note Message

- Pituitary apoplexy represents an uncommon endocrine emergency with potentially life‐threatening status.
- Gonadotropin‐releasing hormone agonist used for prostate cancer has the potential to induce pituitary apoplexy.
- Clinician should ask the patient's past history of pituitary adenoma as well as the other element of MEN.

## 1 Introduction

Gonadotropin‐releasing hormone agonist (GnRHa) is widely used as hormone therapy for prostate cancer. Common adverse events include sexual dysfunction and hot flashes, though headaches rarely occur [1]. Headaches are assumed to derive from pituitary apoplexy. Pituitary apoplexy represents one of the most serious, life‐threatening endocrine emergencies requiring immediate management. It is a clinical syndrome associated with sudden headache, vomiting, and visual impairment caused by the rapid enlargement of a pituitary adenoma.

We report a case of severe headache following GnRHa administration, which led to pituitary apoplexy due to acute enlargement of a pituitary adenoma suspected to be associated with multiple endocrine neoplasia (MEN).

## 2 Case Presentation

A 76‐year‐old male diagnosed with prostate cancer (initial prostate‐specific antigen [PSA]: 6.484 ng/mL; Gleason score: 3 + 3) has been performed as active surveillance due to very low risk group (clinical stage: T1aN0M0) following transurethral resection of prostate.

Five years after PSA elevation, pelvic MRI T2‐weighted and diffusion‐weighted images showed findings suggestive of cancer near the bladder neck. Hormone therapy with GnRHa (leuprolide, 3.6 mg) was initiated. Several hours after the injection, he experienced headache and vomiting at home. In the following evening, he presented to our emergency department with fatigue, convulsive seizures, and inability to open his right eye.

**FIGURE 1:**
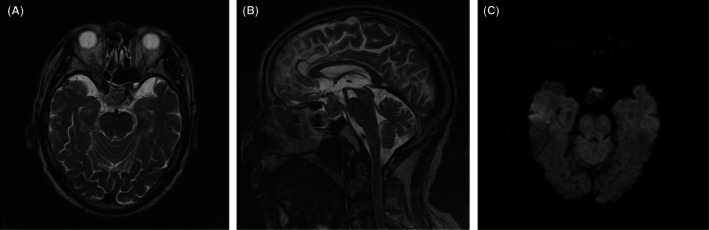
Magnetic resonance imaging of brain. Non‐contrast T2‐weighted axial section (A) coronal section (B), and diffusion‐weighted imaging axial section (C) of pituitary MRI at presentation demonstrating right shifted sellar tumor enlargement followed by hemorrhagic infarction. It is compatible with pituitary apoplexy.

On arrival, right eye movement was restricted to lateral direction. Findings of diplopia and ptosis indicated oculomotor nerve palsy. Brain MRI revealed a hyperintense pituitary adenoma on T2‐weighted image as well as heterogeneously hypointense one on diffusion‐weighted image, shifted to the right and enlarged due to hemorrhagic infarction (Figures 1A–C). Admitted with a diagnosis of pituitary apoplexy, treatment with hydrocortisone 300 mg/day was initiated intravenously. Headaches and nausea gradually improved, allowing tapering of hydrocortisone to 20 mg/day orally, leading to discharge on the 12th day of hospitalization.

One month after GnRHa administration, the dose of hydrocortisone was reduced to a maintenance level. Four months later, the eyelids could open sufficiently, and the size of pituitary gland normalized in brain MRI. Up to now, there has been no recurrence. PSA kinetics shows significant decrease to 0.052 ng/mL 3 months after GnRHa initiation.

We proposed him to restart therapy for prostate cancer. However, he denied any additional therapy due to severe symptoms of pituitary therapy. Thus, we had to wait for his desire to restart it.

## 3 Discussion

GnRHa is one of the representative drugs for hormone therapy in prostate cancer. Headache is a rare adverse event and is resumed to be derived from pituitary apoplexy, though its exact incidence is still unknown.

Pituitary apoplexy refers to hemorrhage or infarction within a pituitary adenoma. It can induce acute neurological symptoms such as sudden headache, visual disturbances (visual acuity, visual field, eye movement), endocrine dysfunction, and altered consciousness due to rapid tumor volume increase [2]. Proper diagnosis and treatment are essential because of life‐threatening symptoms. In 80% of cases of pituitary stroke, the pituitary adenoma is diagnosed only after pituitary stroke [3].

Symptoms are divided into neurological and endocrine manifestations. The most common neurological symptom is sudden severe headache (95%), followed by decreased visual acuity (52%), visual field defects (64%), diplopia (78%), and altered consciousness (5%). In endocrine symptoms, hypopituitarism occupies approximately 80% and secondary acute adrenal insufficiency demonstrates the most important complication [4].

Brain MRI is frequently used for diagnosis; coronal and sagittal T1‐ and T2‐weighted images demonstrate various findings depending on not only the type (hemorrhagic or infarct) but also the timing of onset. Blood tests measuring endocrine hormone levels are performed to assess anterior pituitary function [4, 5].

Although treatment options include conservative management and surgical intervention, conservative management has been recently selected as initial approach. However, when visual impairment due to optic nerve compression is severe, visual function can be hardly resolved. In such cases, surgery should be considered early (ideally within 1 week of symptom onset) [3]. Surgery results in complete or partial improvement of pituitary function in approximately half of the patients [3]. In cases with acute adrenal insufficiency, prompt steroid replacement is crucial.

Regarding pituitary apoplexy following GnRHa administration, Sasagawa et al. [1] compiled 15 cases, all of which presented with severe headache as the initial symptom. Furthermore, among the 15 cases, pathological findings were available for 12 tumors, all of which were LH or FSH producing. Global reports remain below 30 cases, with an average age at the onset of 69.8 years (range 60–85 years). Surgical intervention was performed in 76.2% of cases [6]. Among malignancies, most cases of pituitary apoplexy followed by GnRHa administration are prostate cancer, but only one case is breast cancer [7]. GnRHa was administered as adjuvant therapy after pituitary macroadenoma surgery.

The mechanism of pituitary apoplexy following GnRHa administration remains unclear. However, it is hypothesized that in cases where the pituitary adenoma is an FSH‐ or LH‐producing tumor, stimulation by the GnRHa causes acute tumor enlargement, leading to infarction or hemorrhage [8]. The time to symptom onset after administration typically ranges from 24 to 48 h [6, 9]. With regard to the mechanism, GnRHas, which are lack of an initial gonadotropin surge, might be theoretically preferable in patients with pituitary adenoma.

Pituitary apoplexy may have begun before GnRHa administration. Fortunately, steroid therapy lessened symptoms before visual impairment deterioration, consequently avoiding surgery. Additionally, because of the emergency department visit on the weekend, hormone blood tests were not performed, which made it difficult to predict the adenoma's details.

Pituitary adenoma is one component of MEN Type 1 and is also assumed to be associated with lesions in the parathyroid glands and the pancreatic‐gastrointestinal tract [10]. In our case, a pancreatic islet tumor was noted 5 years prior to GnRHa administration, but the patient denied treatment. GnRHa administration proceeded without considering the existence of MEN. Pretreatment brain CT scan is not routinely recommended before GnRHa injection; a careful collection of past history regarding MEN may help avoid some serious adverse events.

## 4 Conclusion

We report a case of pituitary apoplexy derived from GnRHa administration. Recognizing this adverse event, which remains under‐recognized in urology, is critically important in clinical practice. Particularly given the potential presence of MEN Type 1, it is essential to confirm pituitary, pancreatic, or parathyroid disorders prior to administration.

## Consent

Written informed consent for publication, including images, was obtained from the patient.

## Conflicts of Interest

The authors declare no conflicts of interest.

## Data Availability

The data that support the findings of this study are available on request from the corresponding author. The data are not publicly available due to privacy or ethical restrictions.
